# *Terminalia arjuna*: An overview of its magical properties

**DOI:** 10.6026/9732063002002080

**Published:** 2024-12-31

**Authors:** Vijayalakshmi Rajaram, Ambalavanan Namasivayam, Rajeshkumar Shanmugam, Jaideep Mahendra, Uma Sudhakar, Tharani Munusamy, Uma Subbiah

**Affiliations:** 1 Department of Periodontics, Meenakshi Ammal Dental College and Hospital, Faculty of dentistry, MAHER, Chennai, Tamil Nadu, India; 2 Department of Nanobiomedicine, Centre for Global health research, Saveetha Medical College and Hospital, Saveetha Institute of Medical and Technical Sciences, Saveetha University, Chennai, Tamilnadu, India; 3 Department of Periodontology, Thai Moogambigai Dental College and Hospital, Nerkundram, Chennai, Tamil Nadu, India; 4 Department of Research, MAHER, KK Nagar, Chennai, Tamil Nadu, India

**Keywords:** *Terminalia arjuna*, local drug delivery, periodontitis, herbal agents

## Abstract

*Terminalia arjuna*, native to the Indian subcontinent, is renowned for its cardioprotective benefits, owing to bioactive compounds like antioxidants and anti-inflammatory agents. Beyond heart health, it is gaining attention for applications in oral health, particularly periodontal therapy, due to its anti-inflammatory and wound-healing properties. Its flavonoids and tannins have demonstrated effectiveness in managing bacteria and inflammation in periodontal pockets. This overview explores *Terminalia arjuna*'s phytochemical properties and extensive health benefits beyond cardioprotection, emphasizing its role in oral health management and the importance of sustainable practices in leveraging this ecologically significant plant for innovative periodontal therapies.

## Background:

The natural world has long been a source of potent medicinal remedies, with plants offering a rich repository of bioactive compounds. One such remarkable plant is *Terminalia arjuna*, commonly referred to as Arjuna. Indigenous to the Indian subcontinent, this tree has been revered for centuries in traditional Ayurvedic medicine, particularly for its cardioprotective properties. Arjuna's therapeutic value, however, extends far beyond heart health, with its bark, leaves and fruits being utilized in a wide range of treatments due to its potent antioxidant, anti-inflammatory and antimicrobial activities [1]. In recent years, the growing interest in natural and sustainable approaches to healthcare has renewed attention on *Terminalia arjuna*. Modern scientific studies have not only validated many of the traditional uses of this plant but have also uncovered new potential applications, particularly in the field of periodontal therapy. In periodontal disease management, local drug delivery systems that employ herbal compounds are gaining traction due to their ability to target affected tissues directly while minimizing systemic side effects. *Terminalia arjuna*, with its anti-inflammatory and wound-healing properties, offers immense potential in this area. Its bioactive compounds, such as flavonoids and tannins, have demonstrated efficacy in controlling bacterial colonization and reducing inflammation in periodontal pockets, making it a promising candidate for inclusion in herbal-based local drug delivery systems [2]. Therefore, it is of interest to diverse properties of *Terminalia arjuna*, its phytochemical composition and its wide-ranging benefits, emphasizing its role not only in cardioprotection but also in promoting oral health, particularly in the context of periodontal therapy. Additionally, the environmental sustainability of utilizing herbal-based therapeutics will be discussed, highlighting the plant's significance in both healthcare and ecological management.

## Historical use and ecological distribution:

*Terminalia arjuna* is an evergreen tree in the Combretaceae family that can be found throughout South Asia. The vernacular names of this tree in Indian languages include white marudah, arjuna, arjunam, kakubha and kahu. New leaves typically emerge between February and April, marking the hot season before the tree sheds its foliage. Renowned for its medicinal properties, *T.arjuna* has been a staple in Ayurvedic medicine for over 2,500 years, dating back to the Vedic period. It is extensively referenced in ancient Indian medical texts such as the Charaka Samhita, Sushruta Samhita and Astanga Hridayam. The famous Indian physician Vagbhata was the first to recommend the use of *T.arjuna* stem bark powder for treating heart ailments, further solidifying its importance in traditional medicine [1].

## Phytochemical composition:

*Terminalia arjuna* contains a variety of bioactive compounds that contribute to its therapeutic potential. Key phytochemicals include tannins, which provide antioxidant and anti-inflammatory benefits and flavonoids that support cardiovascular health through lipid-lowering and cardioprotective effects. Additionally, glycosides present in *T.arjuna* offer cardiotonic properties, while triterpenoids, such as ursolic acid, exhibit anticancer and antileishmanial activities. Saponins add further immune-boosting and anti-inflammatory effects and essential minerals complement its overall health benefits. These compounds collectively enhance *T.arjuna*'s potential for use in diverse medical applications, including local drug delivery in periodontal therapy. Major chemical constituents present in it are listed in the Table 1 [3].

## Pharmacological activity:

## Cardiovascular effects:

*Terminalia arjuna* offers a multitude of benefits for cardiac health through various mechanisms at the molecular level. The plant's oleanane triterpenoids, such as arjunic acid, arjunoglycosides, arjunone and arjunolic acid, are primarily responsible for its cardioprotective effects. The bark stem of Arjuna contains diuretic, inotropic and chronotropic effects. Its potent antioxidant activity helps reduce oxidative stress in cardiac cells, thereby preventing damage associated with heart diseases. The herb exhibits anti-apoptotic effects, reducing programmed cell death in cardiac cells during stress conditions like hypoxia, which is facilitated by the regulation of specific signaling pathways. Additionally, *T.arjuna* modulates inflammatory responses by lowering levels of inflammatory cytokines, further supporting cardiac health. It enhances cardiac function by positively influencing calcium handling and myocardial metabolism, which improves contractility. *Terminalia arjuna* extract by alleviating the phosphorylation of JNK and c-jun and also by regulating protein expression levels of Bcl2, Bax, caspase 3, heat shock protein-70 and inducible nitric oxide synthase exhibit antioxidant and anti-apoptotic defense against ischemic-reperfusion injury by enhancing the expression of protective proteins. It also improves lipid profiles by lowering cholesterol levels, thereby reducing the risk of atherosclerosis and other cardiovascular conditions [4, 5].

## Anti-oxidant effect:

Arjunolic acid, a potent triterpene isolated from *Terminalia arjuna*, has been shown to offer substantial cardioprotective effects, primarily by mitigating myocardial necrosis. This protection is attributed to its ability to prevent the depletion of crucial antioxidant enzymes like superoxide dismutase, catalase, glutathione peroxidase (GPO), ceruloplasmin and α-tocopherol. Arjunolic acid also preserves reduced glutathione and ascorbic acid levels, inhibiting lipid peroxidation and myeloperoxidase activity, which play vital roles in combating oxidative stress linked to cardiovascular disorders. Additionally, *T.arjuna*'s arjungenin, another compound from the stem bark, has demonstrated moderate free radical scavenging activity with an IC50 value of 290.6 µg/ml, comparable to that of vitamin C [6]. Terminoside A, an oleanane triterpene and arjunaphthanoloside, a naphthanol glycoside, also inhibit nitric oxide (NO) production in lipopolysaccharide-stimulated macrophages, further contributing to *T.arjuna*'s therapeutic potential in reducing oxidative stress and inflammation associated with heart disease [7].

## Anti-platelet effect:

*Terminalia arjuna* exhibits notable anti-platelet effects, primarily through its active compound arjunolic acid. Studies show that arjunolic acid inhibits thrombin-induced platelet aggregation, achieving an optimal inhibitory concentration (IC50) of 0.048 mM, which is more effective than aspirin's IC50 of 0.088 mM. As the concentration of arjunolic acid increases, the rate of platelet inhibition reaches a steady state, particularly at concentrations above 0.16 mM. This anti-platelet activity is likely due to its ability to modulate the oxidative environment and reduce inflammatory mediators, which are critical in platelet activation and aggregation during cardiovascular diseases. The reduction in platelet aggregation helps prevent thrombus formation, thereby contributing to the cardioprotective properties of *T.arjuna* [8].

## Lipid lowering effect:

Elevated levels of low-density lipoproteins (LDL) and decreased high-density lipoproteins (HDL) are recognized as key risk factors for coronary artery disease. Animal studies have demonstrated that *Terminalia arjuna* bark extract and powder significantly reduce total cholesterol (TC) and triglycerides (TG). The hypolipidemic action is thought to occur through enhanced hepatic clearance of cholesterol, downregulation of lipogenic enzymes and inhibition of HMG-CoA reductase. Specifically, the ethanol extract of *T.arjuna* bark has been found to be effective in reducing LDL cholesterol levels at a dose of 100 mg/kg body weight, with total cholesterol reduction observed at 500 mg/kg body weight [9].

## Anti-hypertensive effect:

*Terminalia arjuna* has shown significant antihypertensive properties, particularly through its ability to improve vascular health. The bark extract induces relaxation of vascular smooth muscles, leading to vasodilation, which helps reduce both systolic and diastolic blood pressure. This effect is partly attributed to its enhancement of endothelial function, which is crucial for maintaining vascular tone. The vasodilatory effects are mediated through increased nitric oxide production, which relaxes the blood vessels and lowers blood pressure. Additionally, *T.arjuna* helps to reduce oxidative stress and inflammation, both of which are contributing factors in the development of hypertension. Its combined antioxidant and anti-inflammatory properties further support its role in managing blood pressure and reducing cardiovascular risk [10].

## Anticancer effect:

*Terminalia arjuna* exhibits significant anti-carcinogenic effects through multiple mechanisms. Its bioactive compounds including flavonoids and triterpenoids provide potent antioxidant activity, which helps reduce oxidative stress, a key factor in cancer initiation. Study done by Verma et al. investigated the effect of aqueous extract of medicinal plant *Terminalia arjuna* on antioxidant defense system in lymphoma bearing AKR mice and showed that arjunolic acid, a key compound in *T.arjuna*, reduced oxidative damage by increasing the activity of important antioxidant enzymes like catalase, superoxide dismutase and glutathione S-transferase in cancer-bearing models [11]. The plant's compounds, particularly arjunolic acid, induce apoptosis selectively in cancer cell without affecting healthy cells. Additionally, *T.arjuna*'s anti-inflammatory properties inhibit the production of pro-inflammatory cytokines and nitric oxide, reducing the chronic inflammation that contributes to cancer progression. The plant also induces cell cycle arrest specifically in G0/G1 phase, slowing down cancer cell proliferation and has shown potential in inhibiting angiogenesis, thereby limiting the blood supply needed for tumor growth [12]. These combined actions make *T.arjuna* a promising candidate for cancer prevention and therapy.

## Anti-inflammatory effect:

*Terminalia arjuna* is recognized for its anti-inflammatory properties, largely attributed to its diverse phytochemical composition. Key compounds include triterpenoids, flavonoids, tannins and glycosides, which play significant roles in mediating inflammation. Studies have demonstrated that extracts from the bark of *T.arjuna* can inhibit the production of nitric oxide (NO) in lipopolysaccharide (LPS)-stimulated macrophages, effectively reducing inflammatory responses [7]. Moreover, certain formulations containing *T.arjuna* have demonstrated enhanced anti-inflammatory and analgesic activities, likely due to the synergistic effects of its phytoconstituents and other herbal components. Arjuna Kaseera Paka (AKP) an ayurvedic formulation of *T.arjuna* produced in cow milk was tested for anti-inflammatory efficacy and compared to a hydroalcoholic extract. The study found that AKP has better efficacy, maybe due to the presence of milk solids. Milk solids enhanced the absorption of *T.arjuna* phytoconstituents, resulting in better effectiveness even at lower medication concentrations [13]. This collective action underscores the plant's potential as a natural anti-inflammatory agent, supporting its traditional use in Ayurvedic medicine.

## Anti-bacterial effect:

*Terminalia arjuna* has demonstrated significant antibacterial activity against various pathogens. Research showed that extracts from its bark, leaves, roots and fruits exhibited antibacterial effects on both Gram-positive and Gram-negative bacteria. In particular, studies found that the aqueous extracts were effective in limiting the growth of tested microbial strains, although the sensitivity varied based on the plant part and the bacterial species involved [14]. In addition to its notable antibacterial properties, *Terminalia arjuna* was found to possess a range of phytochemicals that contribute to its efficacy. The major bioactive constituents included tannins, flavonoids and phenolic compounds, which were known for their antimicrobial properties. These compounds interfered with bacterial cell walls, leading to cell lysis and death, thereby effectively combating infections. The findings suggested that *T.arjuna*'s phytochemical components could serve as potential antimicrobial agents, supporting its traditional use in medicine.

## Wound healing effect:

*Terminalia arjuna* exhibited significant wound healing activity due to its rich phytochemical composition, particularly tannins. Tannins from the bark can significantly reduce wound size and enhance tissue tensile strength. The herb accelerated wound healing by promoting collagen turnover, essential for skin repair. Traditionally, the ground bark is applied topically to enhance healing outcomes. *T.arjuna* bark alcoholic extract was applied to rat cutaneous wounds using in vivo methods to examine its wound healing potential. The study found that positive impact was linked to the tannin concentration [15]. Himax ointment and lotion with *T.arjuna* extract demonstrated equivalent wound healing capacity to the conventional medication, nitrofurazone [16]. Overall, these findings underscore the therapeutic potential of *Terminalia arjuna* as an effective natural alternative for wound healing, highlighting its efficacy in promoting faster recovery and enhancing healing outcomes compared to conventional treatments.

## Therapeutic uses:

*Terminalia arjuna*, a well-regarded herb in traditional medicine, exhibits a broad spectrum of therapeutic applications, particularly in cardiovascular health and beyond.

[1] Cardiovascular Health: *T.arjuna* is predominantly recognized for its cardioprotective properties. Clinical studies demonstrate that its bark powder can effectively reduce the frequency of anginal attacks and improve overall cardiac function in patients with stable angina and coronary artery disease. Administration of 500 mg of the extract resulted in significant improvements in exercise tolerance as well as reductions in systolic blood pressure, plasma cortisol and serum cholesterol levels. The extract of *T. arjuna* exhibits antithrombotic properties, contributing to reduced platelet aggregation in patients with coronary artery disease. This effect may play a crucial role in preventing thrombotic events [17].

[2] Management of Congestive Heart Failure (CHF): Research suggests that *T.arjuna* can improve the quality of life for CHF patients. In clinical trials, patients taking 4 g of bark powder daily reported improved functional status, increased diuresis and reductions in both systolic and diastolic blood pressure. Long-term follow-ups revealed sustained benefits in left ventricular function [18].

[3] Rheumatic Heart Disease: *T.arjuna* has demonstrated efficacy in managing rheumatic heart disease. In double-blind studies, *T. arjuna* causes significant improvements in left ventricular ejection fraction and functional capacity [19].

[4] Ischemic Mitral Regurgitation: In cases of ischemic mitral regurgitation post-myocardial infarction, *T.arjuna* has been shown to significantly decrease the severity of the condition and enhance diastolic function, leading to better overall cardiac performance [20].

[5] Cardiomyopathy: The herb has also been studied for its role in treating cardiomyopathy. Observational studies suggest that when combined with standard therapies, *T.arjuna* leads to notable improvements in left ventricular ejection fraction and functional capacity [21].

[6] Oxidative Stress and Dyslipidemia: Studies highlight the antioxidant properties of *T.arjuna*, showing its potential to improve lipid profiles significantly. It has been found to lower total cholesterol (TC), low-density lipoprotein (LDL) and triglyceride (TG) levels while raising high-density lipoprotein (HDL) levels, thus mitigating cardiovascular risk factors. In a prospective cohort research, dyslipidemic patients were given *T.arjuna* powder (5 g, BD) for three weeks, followed by Arogyavardhini Vati (500 mg, BD) for four weeks. There was a significant reduction in TC, LDL, TG, serum C-reactive protein, blood glucose and an increase in HDL levels, supporting the significance of *T.arjuna* in dyslipidemic patients [22].

[7] Endothelial Function: Research involving smokers indicated that *T.arjuna* can positively affect endothelial health, leading to significant improvements in endothelial dysfunction [23].

## Other health benefits:

*Terminalia arjuna* is a versatile herb known for its numerous health benefits beyond cardiovascular applications. It has demonstrated significant wound healing properties due to its rich phytochemical composition, particularly tannins, which enhance collagen synthesis and improved tissue tensile strength. Additionally, *T.arjuna* shows promise in diabetes management by aiding glycemic control and improving insulin sensitivity [24]. Its anti-inflammatory effects are beneficial in managing conditions like arthritis, while its antioxidant properties combat oxidative stress, potentially preventing chronic diseases. The herb also supports digestive health by alleviating gastrointestinal disorders and its expectorant properties benefit respiratory conditions. Furthermore, *T.arjuna* exhibits antimicrobial activity, making it effective against various pathogens. Collectively, these diverse therapeutic applications underscore the potential of *Terminalia arjuna* as a valuable natural remedy in holistic health approaches [2].

## Uses in periodontal therapy:

*Terminalia arjuna* has garnered significant attention in the field of periodontal therapy due to its beneficial properties in managing periodontal diseases. The plant is rich in bioactive compounds such as flavonoids, tannins and triterpenoids, which contribute to its anti-inflammatory, antimicrobial and wound-healing effects. These properties make it a promising candidate for local drug delivery systems aimed at treating periodontal conditions. In a double blind randomized controlled study done to evaluate the efficacy of a Terminalia chebula 10% mouth rinse compared with chlorhexidine 0.12% mouth rinse, applied two times daily for 2 weeks, in the treatment of dental plaque and gingivitis, the results demonstrated Terminalia chebula mouth rinse was effective in reducing microbial plaque, gingival inflammation and neutralizing salivary pH [25]. In an in-vitro study the antiadhesive property of herbal extracts was evaluated using Glycyrrhiza glabra (GG) and Terminalia chebula (TC) herbal extracts on Streptococcus mutans. The results showed both herbal extracts have significant antiadhesive and antimicrobial activity against *S. mutans*, however, high antiadherence property was seen with TC than GG [26].

## Toxicity and side effects:

*Terminalia arjuna* is generally considered safe for medicinal use; however, some studies have reported potential toxicity and side effects associated with its consumption. The most commonly observed adverse effects include gastrointestinal disturbances, such as diarrhea, nausea and abdominal pain. High doses may lead to hepatotoxicity, as indicated in certain animal studies that demonstrated increased liver enzymes. Additionally, *T.arjuna* may interact with other medications, particularly anticoagulants, due to its potential antithrombotic properties, which could increase the risk of bleeding. Individuals with existing liver or kidney conditions should exercise caution and consult healthcare professionals before using T. arjuna, as its effects on these organs require further investigation. Given the varying compositions of herbal preparations, individual responses can differ significantly, necessitating the importance of dosage standardization and careful monitoring during use. More extensive clinical trials are required to establish a comprehensive understanding of the safety profile of *T. arjuna*, including long-term effects and potential interactions with other medications [3].

## Sustainability and conservation:

Sustainable harvesting practices are crucial for preserving *Terminalia arjuna* and ensuring its availability for future generations. Responsible collection methods, such as selective harvesting and avoiding over-exploitation, can help maintain healthy populations in their natural habitats. However, *T.arjuna* faces several threats, including habitat destruction due to deforestation, urbanization and agricultural expansion, which jeopardize its survival. Additionally, climate change poses a significant risk, altering the ecological balance required for its growth. To mitigate these threats, conservation strategies must be implemented, including the establishment of protected areas, reforestation initiatives and community-based conservation programs that involve local populations in sustainable management practices. Educating communities about the ecological and medicinal importance of *T.arjuna* can foster a sense of stewardship and promote conservation efforts. Furthermore, supporting research on its ecological requirements and developing cultivation techniques can enhance the sustainability of *T.arjuna* outside its natural habitat. Overall, a multifaceted approach combining sustainable practices, habitat protection and community engagement is essential to ensure the conservation of *Terminalia arjuna* for its continued medicinal and ecological contributions.

## Future directions:

Despite the promising therapeutic potential of *Terminalia arjuna*, several knowledge gaps warrant further investigation. Mechanistic studies are needed to elucidate the specific biochemical pathways and molecular mechanisms through which *T.arjuna* exerts its effects, particularly in cardiovascular health and diabetes management. Standardization of extraction and formulation protocols is essential to ensure consistency in the composition and efficacy of *T.arjuna* products across various studies. Long-term studies evaluating the safety and effectiveness of chronic use in diverse patient populations, especially those with comorbidities, are crucial. Additionally, comparative effectiveness research should focus on direct comparisons between *T.arjuna* and conventional pharmacological treatments to identify potential synergistic benefits. Rigorous randomized controlled trials are necessary to establish clear clinical guidelines for its use in various health conditions. Understanding potential drug interactions is also vital, particularly for patients requiring polypharmacy. Furthermore, exploring broader therapeutic applications, including its role in cancer treatment and neuroprotection, could expand its utility in modern medicine. Population-specific studies, along with research on the bioavailability and pharmacokinetics of its compounds, will enhance our understanding of *T.arjuna*. Finally, ethnobotanical studies investigating traditional uses across different cultures may uncover additional therapeutic applications, further informing contemporary clinical practices. Addressing these gaps can significantly contribute to the evidence supporting the use of *Terminalia arjuna* in healthcare.

## Conclusion:

*Terminalia arjuna* is a valuable medicinal plant with a rich phytochemical profile, showing therapeutic promise in cardiovascular health, diabetes management, wound healing and periodontal therapy, due to its anti-inflammatory and antimicrobial properties. Addressing existing knowledge gaps through rigorous research on its mechanisms, safety and comparative effectiveness with conventional treatments is crucial to fully realize its benefits. Sustainable harvesting and conservation strategies are essential to preserve *T.arjuna* from threats like habitat loss and climate change, necessitating collaboration among researchers, healthcare practitioners and local communities.

## Figures and Tables

**Table 1:** Phytochemical composition of *Terminalia arjuna*

**Parts of plant**	**Major chemical constituent**	**Major chemical constituent**
Stem Bark	Triterpenoids	Arjunin, arjunic acid, arjunolic acid, arjungenin, terminic acid, ajung IV and V, arjunasides A-E, 2-α, 3-β dihydroxyurs-12,18-dien-28-O-beta-d-glucopyranosyl ester
	Glycosides	Arjunetin, arjunoside I, arjunoside II, arjunaphthanoloside, terminosi
	Flavonoids	Arjunolone, arjunone, baicalein, luteolin, gallic acid, ethyl gallate, qu pelargonidin, oligomeric proanthocyanidins
	Tannins	Pyrocatechols, punicallin, punicalagin, terchebulin, terflavin C, casta
	Minerals/trace elements	Calcium, aluminum, magnesium, silica, zinc, copper
Roots	Triterpenoids	Arjunic acid, arjunolic acid, oleanolic acid, terminic acid
	Glycosides	Arjunoside I, arjunoside Il, arjunoside III, arjunoside IV, 2a, 19a-dihy 28-oic Acid 28-O-B-d-glucopyranoside
Leaves	β-sitosterol	
	Flavonoids	
	Alkaloids	
	Tannins	
